# Prognostic role of the pathological status following neoadjuvant chemoradiotherapy and surgery in esophageal squamous cell carcinoma

**DOI:** 10.1186/s12885-025-13465-w

**Published:** 2025-01-10

**Authors:** Xiaofeng Duan, Jie Yue, Shangren Wang, Fangdong Zhao, Wencheng Zhang, Shuo Qie, Hongjing Jiang

**Affiliations:** 1 Department of Minimally Invasive Esophageal Surgery, Key Laboratory of Cancer Prevention and Therapy, Tianjin Medical University Cancer Institute & Hospital, National Clinical Research Center for Cancer, Tianjin’s Clinical Research Center for Cancer, Tianjin, China; 2 Department of Radiation Oncology, Key Laboratory of Cancer Prevention and Therapy, Tianjin Medical University Cancer Institute & Hospital, National Clinical Research Center for Cancer, Tianjin’s Clinical Research Center for Cancer, Tianjin, 300060 China; 3 Department of Pathology, Key Laboratory of Cancer Prevention and Therapy, Tianjin Medical University Cancer Institute & Hospital, National Clinical Research Center for Cancer, Tianjin’s Clinical Research Center for Cancer, Tianjin, 300060 China; 4 Department of Minimally Invasive Esophageal Surgery, Key Laboratory of Cancer Prevention and Therapy, Tianjin Medical University Cancer Hospital and Institute, National Clinical Research Center for Cancer, Tianjin’s Clinical Research Center for Cancer, Tiyuanbei, Huanhuxi Rd., Hexi District, Tianjin, 300060 China

**Keywords:** Neoadjuvant therapy, Chemoradiotherapy, Esophageal squamous cell carcinoma, Esophagectomy

## Abstract

**Background:**

In this study, we retrospectively examined the prognostic significance of the pathological status of esophageal squamous cell carcinoma (ESCC) patients following neoadjuvant chemoradiotherapy (NCRT) and surgery.

**Methods:**

Data of patients with cT2-4aN0-3 stage ESCC who underwent NCRT and esophagectomy during 2014–2022 were reviewed retrospectively. Survival differences were compared according to revised TN (rTN) stage (ypT0N0, ypT + N0, ypT0N+, and ypT + N+) using univariate and Cox regression analyses.

**Results:**

Of the 136 patients (59.1 ± 7.2 y) included in this study, 123 (90.4%) were males. There were 39 (28.7%) patients with ypT0N0 disease, 49 (36.0%) ypT + N0, 11 (8.1%) ypT0N+, and 37 (27.2%) ypT + N+. Additionally, 126 patients had a median follow-up period of 30 (1–90) months. The 5-year overall survival was 81.6% in ypT0N0 group, 53.1% for ypT + N0, 50.0% for ypT0N+, and 18.6% for ypT + N+ (*p* < 0.001) and 5-year disease-free survival was 70.1% for ypT0N0, 39.7% for ypT + N0, 33.3% for ypT0N+, and 18.4% for ypT + N+ (*p* < 0.001). The ypT + N0 and ypT0N + groups showed no significant differences in survival (*p* > 0.05). In Cox regression analysis, ypT stage and rTN stage showed an independent association with OS (*p* = 0.026 and 0.001, respectively). During the follow-up period, 69 (54.8%) patients developed recurrence, with ypT0N0 patients experiencing fewer local and distant recurrences compared to other groups (*p* < 0.001).

**Conclusion:**

In ESCC patients, the ypT0N0 status after NCRT predicts prolonged survival, but this reduces significantly when nodal metastases or residual primary lesions are present.

## Introduction

Being a prevalent malignant tumor worldwide, esophageal cancer (EC) ranks seventh in incidence and sixth in cancer-related mortality, with approximately 604,100 and 544,076 new cases and fatalities reported in 2021, respectively [1]. East Asian countries like China show a high incidence of esophageal squamous cell carcinoma (ESCC). EC treatment has evolved from a single modal to a multimodal approach over the past decade. According to the NEOCRTEC5010 and CROSS studies [2–4], the standard treatment of neoadjuvant chemoradiotherapy (NCRT) plus surgery offers significant survival benefits to EC patients with locally advanced ESCC.

In EC, pathological complete response (pCR), indicated as ypT0N0, is characterized by the absence of cancer in the primary esophagus and lymph nodes (LNs). It is a clinically significant parameter for evaluating the treatment response to NCRT and predicting survival outcomes. Patients with ESCC achieving pCR had prolonged survival, with a 5-year overall survival (OS) rate of 79.3% and disease-free survival (DFS) rate of 77%, compared to 54.8% and 51.2%, respectively, in those who did not achieve pCR [5]. For non-pCR patients, there are three different pathological statuses based on the residual tumor in the esophagus or LNs: ypT0N+, ypT + N0, and ypT + N+. The prevalence and prognostic value of other pathological states have been rarely elucidated [6, 7].

Here, we examined the prognostic value of pathological responses in both primary tumors and LNs of advanced-stage ESCC patients. We compared the survival and recurrence patterns based on the revised TN (rTN) stage (ypT0N0, ypT + N0, ypT0N+, and ypT + N+) of patients who received NCRT plus surgery.

## Methods

### Patients

This study included all the ESCC patients who received NCRT plus esophagectomy at the cancer center during May 2014 and April 2022. Detailed information on preoperative evaluation and surgery are provided in our previous study [8]. ESCC patients who received NCRT plus a three-incision esophagectomy (McKeown procedure) were included in this study, while patients who showed non-SCC pathology, underwent Ivor-Lewis surgery, or underwent preoperative chemotherapy, immunotherapy, or surgery alone were excluded from this analysis.

### Preoperative examination

The routine preoperative examination included endoscopic biopsy and endosonography. Cervival ultrasonography and thoracic and abdominal enhanced scanning computed tomography (CT) were performed to determine the local growth, lymph node and distant metastasis. The positron emission tomography (PET) was only used in some cases to exclude metastatic diseases and evaluate resectability.

### Chemoradiotherapy

Chemotherapy was conducted once a week using platinum-based agents paired with fluorouracil, docetaxel, or paclitaxel. Meanwhile, radiotherapy (40–41.4 Gy; 20‒23 fractions and 1.8–2.0 Gy per fraction) was conducted for 5 days a week. The radiation dose was calculated using image-guided or intensity-modulated radiotherapy. Gross tumor volume (TV) included the esophageal tumor along with enlarged locoregional LNs; clinical TV encompassed a 6-mm radial margin and 3-mm distal and proximal margins around the gross TV to account for subclinical involvement; and planning TV consisted of a 5-mm margin around the clinical TV to account for setup variations and tumor motion.

### Surgical procedure

Surgical procedures comprised left cervical esophagogastric anastomosis, gastric tube construction, stomach mobilization, LN dissection, and transthoracic esophageal dissection. Minimally invasive esophagectomy (MIE) was performed through robot- and video-assisted approaches. In this study, all patients underwent two field lymph node dissection. For patients with upper esophageal cancer or preoperative examination indicating suspicious cervical lymph node metastasis, three field lymph node dissection was performed. LN dissection was performed around the bilateral recurrent laryngeal nerves during the thoracic procedure, which has been discussed in detail in our previous studies [8, 9].

### Data collection

Collected data included patient demographics; cT, cN, and cTNM stages; neoadjuvant treatment details (including chemotherapy regimen and radiotherapy dosage); the period between surgery and neoadjuvant therapy (NAT); surgical factors (approach, operative time, and blood loss); hospital stay post-surgery; morbidity and mortality post-surgery; pathology results; adjuvant therapy; and follow-up information. Pathology reports recorded the number of harvested LNs. Residual viable tumor of ≤ 10% in the primary lesion was defined as major pathological response (MPR). For esophageal tumors, tumor regression grade (TRG) was categorized as T1–4 for 0%, ≤ 10%, ≤ 50%, and < 100% residual tumor, respectively and T5 for no tumor regression. Operative duration was measured from the initial incision to the final closure, as well as the time spent on repositioning the body during the operation. OS was measured from the date of initiation of NAT to the date of death due to any cause. For the surviving patients, censoring was conducted on the date of their most recent follow-up. The DFS was measured from the surgery date to the date of disease recurrence or death. Disease recurrence was classified as either locoregional (affecting the esophageal bed or regional LNs) or distant (affecting distant LNs or organs like brain, lungs, bones, and liver) disease. The Esophagectomy Complication Consensus Group criteria were used to classify all the significant complications [10], and the 8th edition of American Joint Committee on Cancer Tumor-Node-Metastasis Staging System was used for staging the patients [11].

### Statistical analyses

Continuous variables are displayed as mean ± standard deviation and analyzed by Wilcoxon rank-sum test or unpaired student’s *t*-test. Categorical variables are reported as frequencies (%) and analyzed by Fisher’s exact test or chi-squared test. The Kaplan–Meier method was used to plot OS and DFS curves, and the log-rank test was used to compare the curves. The prognostic risk factors were determined by univariate and Cox regression analyses. Only variables with statistical differences in univariate analysis (*p* < 0.05) were subjected to multivariate analysis. Statistical analyses were performed using SPSS v25 (IBM SPSS Statistics, Windows, version 25.0, USA) at a significance value of *p* < 0.05.

## Results

### Patient demographics

In this study, we analyzed 136 patients (59.1 ± 7.2 y), of which 123 (90.4%) were males. Table 1 summarizes the baseline characteristics of the patients based on their pathological responses. Based on the pathological status, 28.7% (*n* = 39) of the patients were classified as ypT0N0 (pCR), with the remaining categorized as ypT + N0 (49/136, 36.0%), ypT0N+(11/136, 8.1%), and ypT + N+(37/136, 27.2%). A major pathological response (MPR) to the primary tumor was observed in 76 patients (55.9%).

**Table 1:** Demographics variables

	All patients, *n* = 136 (%)	ypT0N0, *n* = 39 (%)	ypT + N0, *n* = 49 (%)	ypT0N+, *n* = 11 (%)	ypT + N+, *n* = 37 (%)	*P* value
Age, years, mean ± SD	59.1 ± 7.2	59.6 ± 7.0	56.0 ± 7.7	58.2 ± 7.3	60.2 ± 7.3	0.415
Sex ratio (M: F)	123:13	32:7	45:4	10:1	36:1	0.151
Smoking (n, %)	107 (78.7)	26 (66.7)	40 (81.6)	10 (90.9)	31 (83.8)	0.201
Drinking (n, %)	105 (77.2)	23 (59.0)	41 (83.7)	9 (81.8)	32 (86.5)	0.020
Comorbidity						
Hypertention	33 (24.3)	11 (28.2)	11 (22.4)	5 (45.5)	6 (16.2)	0.231
Diabetes	10 (7.4)	2 (5.1)	7 (14.3)	0	1 (2.7)	0.192
Coronary heart disease	6 (4.4)	1 (2.6)	2 (4.1)	2 (18.2)	1 (2.7)	0.200
Tumor location						0.365
20–25 cm	5 (3.7)	1 (2.6)	3 (6.1)	1 (9.1)	0	
>25 & <=30 cm	61 (44.9)	21 (53.8)	22 (44.9)	5 (45.5)	13 (35.1)	
>30 cm	70 (51.5)	17 (43.6)	24 (49.0)	5 (45.5)	24 (64.9)	
cT stage						0.216
T2	3 (2.2)	3 (7.7)	0	0	0	
T3	74 (54.4)	21 (53.8)	27 (55.1)	7 (63.6)	19 (51.4)	
T4a	59 (43.4)	15 (38.4)	22 (44.9)	4 (36.4)	18 (48.6)	
cN stage						0.339
N0	30 (14.3)	10 (13.5)	20 (14.7)	9 (13.6)	9 (13.6)	
N1	103 (48.1)	36 (46.2)	67 (49.3)	34 (51.5)	30 (45.5)	
N2	70 (33.3)	30 (40.5)	40 (29.4)	17 (25.8)	25 (37.9)	
N3	11 (5.2)	2 (2.7)	9 (6.6)	6 (9.1)	2 (3.0)	
cTNM stage						0.563
II	10 (7.4)	4 (10.3)	2 (4.1)	2 (18.2)	2 (5.4)	
III	60 (44.1)	19 (48.7)	23 (46.9)	4 (36.4)	14 (37.8)	
IV	66 (48.5)	16 (41.0)	24 (49.0)	5 (45.5)	21 (56.8)	
Surgical procedure						0.002
Open	41 (30.1)	4 (10.3)	19 (38.8)	2 (18.2)	16 (43.2)	
Video-assisted MIE	65 (47.8)	18 (46.2)	24 (49.0)	7 (63.6)	16 (43.2)	
Robot-assisted MIE	30 (22.1)	17 (43.6)	6 (12.2)	2 (18.2)	5 (13.5)	
Year						0.156
2014–2016	49 (36.0)	9 (23.1)	17 (34.7)	5 (45.5)	18 (48.6)	
2017–2019	70 (51.5)	21 (53.8)	27 (55.1)	6 (54.5)	16 (43.2)	
2020–2022	17 (12.5)	9 (23.1)	5 (10.2)	0	3 (8.1)	
Lymph node dissection						0.622
3-field	13 (9.6)	3 (7.7)	4 (8.2)	2 (18.1)	4 (10.8)	
2-field	123 (90.4)	36 (92.3)	45 (91.8)	9 (81.8)	33 (89.2)	
Operation time, mins, mean ± SD	311.4 ± 61.0	305.5 ± 58.8	311.0 ± 62.0	319.1 ± 71.3	315.9 ± 61.2	0.880
Surgical blood loss, ml, mean ± SD	188.9 ± 75.2	168.6 ± 61.9	205.9 ± 76.5	139.0 ± 76.1	201.4 ± 77.1	0.015
Surgical interval, days, mean ± SD	47.6 ± 12.1	48.2 ± 12.5	47.5 ± 11.5	47.0 ± 8.6	47.4 ± 13.5	0.990
Adjuvant chemotherapy	64 (47.1)	11 (28.2)	28 (57.1)	5 (45.5)	20 (54.1)	0.040

After NCRT, more ypT0N0 patients (89.8%) underwent minimally invasive surgery (MIE), and more patients with primary tumor residues (ypT + N0 38.8% and ypT + *N* + 43.2%, respectively) underwent open procedure (*p* = 0.002). Surgical blood loss was greater in the ypT + N0 and ypT + N + groups compared to the ypT0N0 and ypT0N + groups (*p* = 0.015). Regarding the adjuvant therapy, ypT0N0 was least frequently underwent after surgery (*p* = 0.048). The revised TN (rTN) stage was related with ypT status, ypN status, ypStage, and TRG grade (*p* < 0.001; Table 2).

**Table 2:** Pathological and clinical outcomes of four groups

	All patients, *n* = 136 (%)	ypT0N0, *n* = 39 (%)	ypT + N0, = 49 (%)	ypT0N+, *n* = 11 (%)	ypT + N+,*n* = 37 (%)	*P* value
Lymph node harvest, mean ± SD	21.0 ± 9.9	21.9 ± 11.6	18.3 ± 7.2	20.5 ± 7.8	23.6 ± 10.9	0.087
Metastatic lymph node, mean ± SD	0.8 ± 1.8	0	0	1.6 ± 1.2	2.6 ± 2.5	< 0.001
ypT stage						< 0.001
T0	50 (36.8)	39 (100)	0	11 (100)	0	
T1	12 (8.8)	0	8 (16.3)	0	4 (10.8)	
T2	22 (16.2)	0	15 (30.6)	0	7 (18.9)	
T3	38 (27.9)	0	19 (38.8)	0	19 (51.4)	
T4	14 (10.3)	0	7 (14.3)	0	7 (18.9)	
ypN stage						< 0.001
N0	88 (64.7)	39 (100)	49 (100)	0	0	
N1	37 (27.2)	0	0	10 (90.9)	27 (73.0)	
N2	7 (5.1)	0	0	1 (9.1)	6 (16.2)	
N3	4 (2.9)	0	0	0	4 (10.8)	
ypTNM stage						< 0.001
I	62 (45.6)	39 (100)	23 (46.9)	0	0	
II	19 (14.0)	0	19 (38.8)	0	0	
III	38 (27.9)	0	7 (14.3)	10 (90.9)	21 (56.8)	
IV	17 (12.5)	0	0	1 (9.1)	16 (43.2)	
Major pathological response	76 (55.9)	39 (100)	18 (36.7)	11 (100)	8 (21.6)	< 0.001
TRG grade, primary tumor						< 0.001
1 (no residual tumor cell)	50 (36.8)	39 (100)	0	11 (100)	0	
2 (residual tumor ≤ 10%)	26 (19.1)	0	18 (36.7)	0	8 (21.6)	
3 (residual tumor ≤ 50%)	13 (9.6)	0	7 (14.3)	0	6 (16.2)	
4 (residual tumor < 100%)	15 (11.0)	0	5 (10.2)	0	10 (27.0)	
5 (no tumor regression)	32 (23.5)	0	19 (38.8)	0	13 (35.1)	
Follow-up, cases	126	38	43	10	35	-
median (range), months	30 (1–90)					
Death at last follow-up	57 (44.9)	6 (15.8)	19 (43.2)	5 (50)	27 (77.1)	< 0.001
Recurrence at last follow-up	69 (54.8)	10 (26.3)	24 (55.8)	7 (70)	28 (80)	< 0.001
Recurrence location						< 0.001
locoregional	16 (12.7)	2 (5.3)	5 (11.6)	1 (10.0)	8 (22.9)	
Distant metastasis	22 (17.5)	6 (15.8)	4 (9.3)	6 (60.0)	6 (17.1)	
Local + distant	6 (4.8)	0	5 (11.6)	0	1 (2.9)	
Death	21 (16.7)	2 (5.3)	6 (14.0)	0	13 (37.1)	
No details	4 (3.2)	0	4 (9.3)	0	0	

### Recurrence pattern

Of the 136 patients, 10 were lost during follow-up. The remaining 126 patients had a median follow-up period of 30 (1–90) months. As of the last follow-up, 69 patients (54.8%) remained alive, while 57 (45.2%) had passed away. Additionally, at the time of the final follow-up, 57 patients (45.2%) showed no clinical signs of tumor recurrence, while 69 (54.8%) either experienced disease recurrence or death. The locoregional recurrence and distant metastasis rates were 12.7% (*n* = 16) and 17.5% (*n* = 22), and six patients (4.8%) had both local and distant metastases. The ypT0N0 patients showed an overall recurrence rate (locoregional and/or distant) of 26.2% (10/39), significantly lower than the rates observed in the other three groups (55.8% for ypT + N0, 70% for ypT0N+, and 80% for ypT + N+, *p* < 0.001). The ypT0N0 patients had fewer local and distant recurrences than the other groups (*p* < 0.001). Table 2 summarizes the recurrence rates of the four groups.

### Survival date

**Fig. 1:**
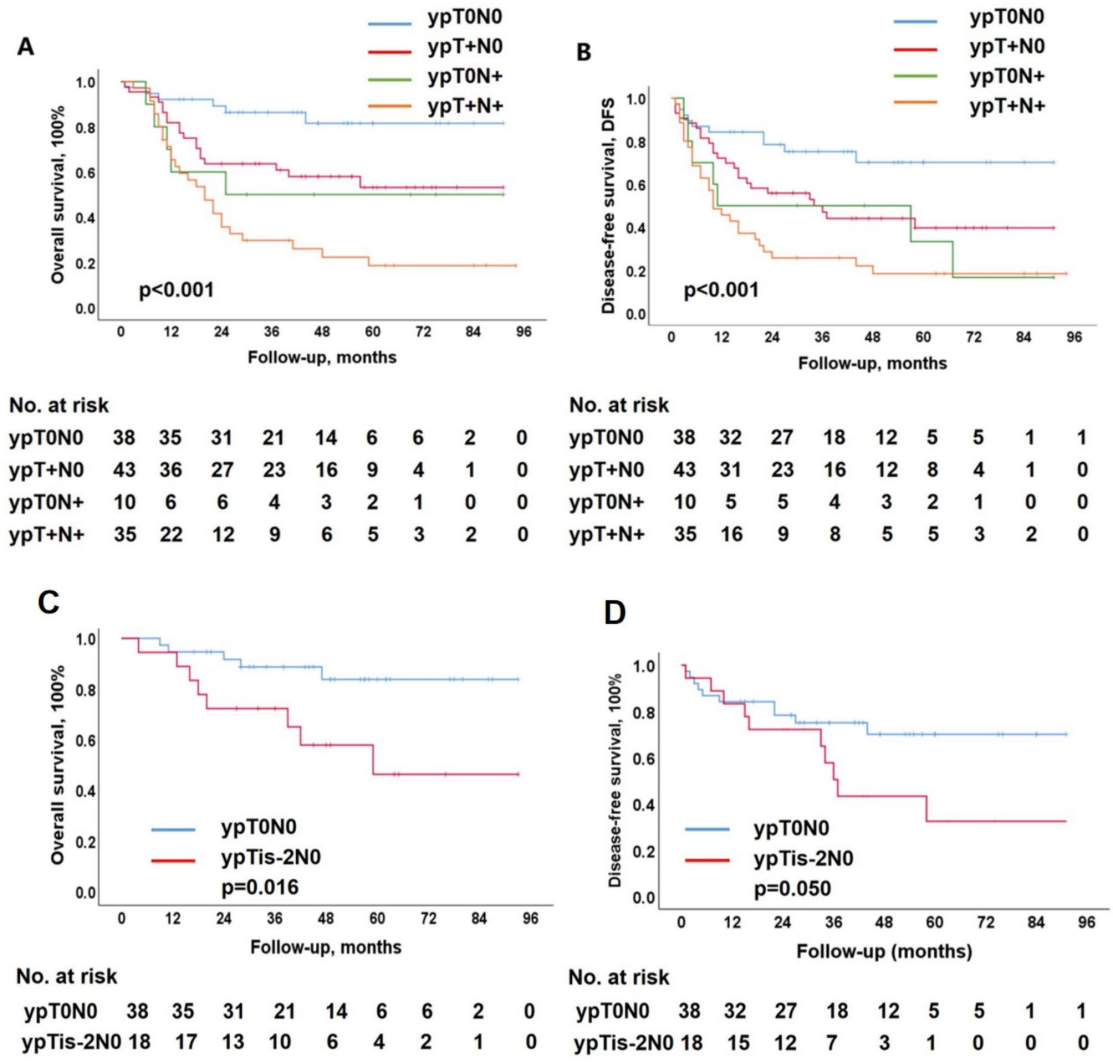
Long-term survival according to revised TN stage: ypT0N0, ypT + N0, ypT0N + and ypT + N+

Among the 126 patients with follow-up data, the 1-year OS was 78.7%, 3-year OS was 73.9%, and 5-year OS was 50.4%. Based on the pathological status, the ypT0N0, ypT + N0, ypT0N+, and ypT + N + groups showed 5-year OS of 81.6%, 53.1%, 50.0%, and 18.6%, respectively (*p* < 0.001) (Fig. 1A). Among these, the ypT + N + group had the poorest OS, while the ypT0N0 group exhibited the highest survival following NCRT and surgery. Meanwhile, the ypT0N + group exhibited a similar OS to the ypT + N0 group (*p* > 0.05).

Among the 126 patients with follow-up data, the 1-year DFS was 66.7%, 3-year OS was 49.6%, and 5-year OS was 41.3%. Based on the pathological status, the ypT0N0, ypT + N0, ypT0N+, and ypT + N + groups showed 5-year DFS of 70.1%, 39.7%, 33.3%, and 18.4%, respectively (*p* < 0.001) (Fig. 1B). Among these, the ypT + N + group had the poorest DFS, while the ypT0N0 group had the highest DFS following NCRT and surgery. The ypT0N + group exhibited a similar DFS to that of the ypT + N0 group (*p* > 0.05).

### Discrepancy based on AJCC 8th ypStage

The 8th of AJCC manual classifies patients with ypT0N0 and other ypTis-2N0 (ypT + N0) statuses as ypStage I, although patients with ypT0N0 status exhibit markedly higher OS (*p* = 0.016; Fig. 1C) and DFS (*p* = 0.050; Fig. 1D) compared to the patients with ypTis-2N0 status. Another discrepancy was observed between ypT1-2N0 (ypT + N0) and ypT0N1-2 (ypT0N+). Patients with ypT1-2N0 status are classified as ypStage I, while those with ypT0N1-2 status are classified as ypstage IIIA, according to the AJCC system, and both the groups showed no significant differences in their 5-year OS (46.2% vs. 50%, *p* = 0.616) and DFS rates (32.5% vs. 33.3, *p* = 0.481).

### Influences of pathological status on survival

**Fig. 2:**
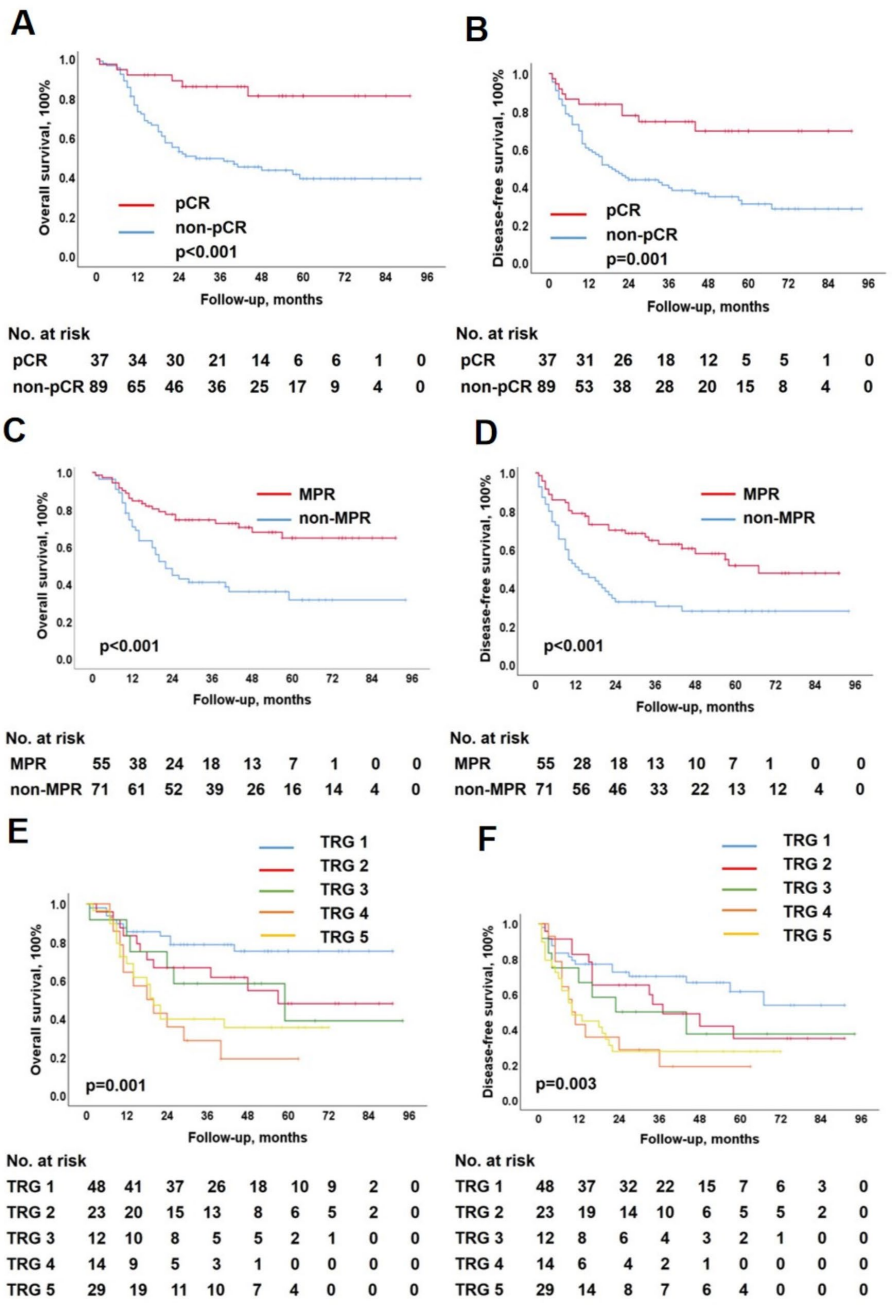
Long-term survival according to different pathological status: pCR, MPR and TRG grade of primary tumor

**Fig. 3:**
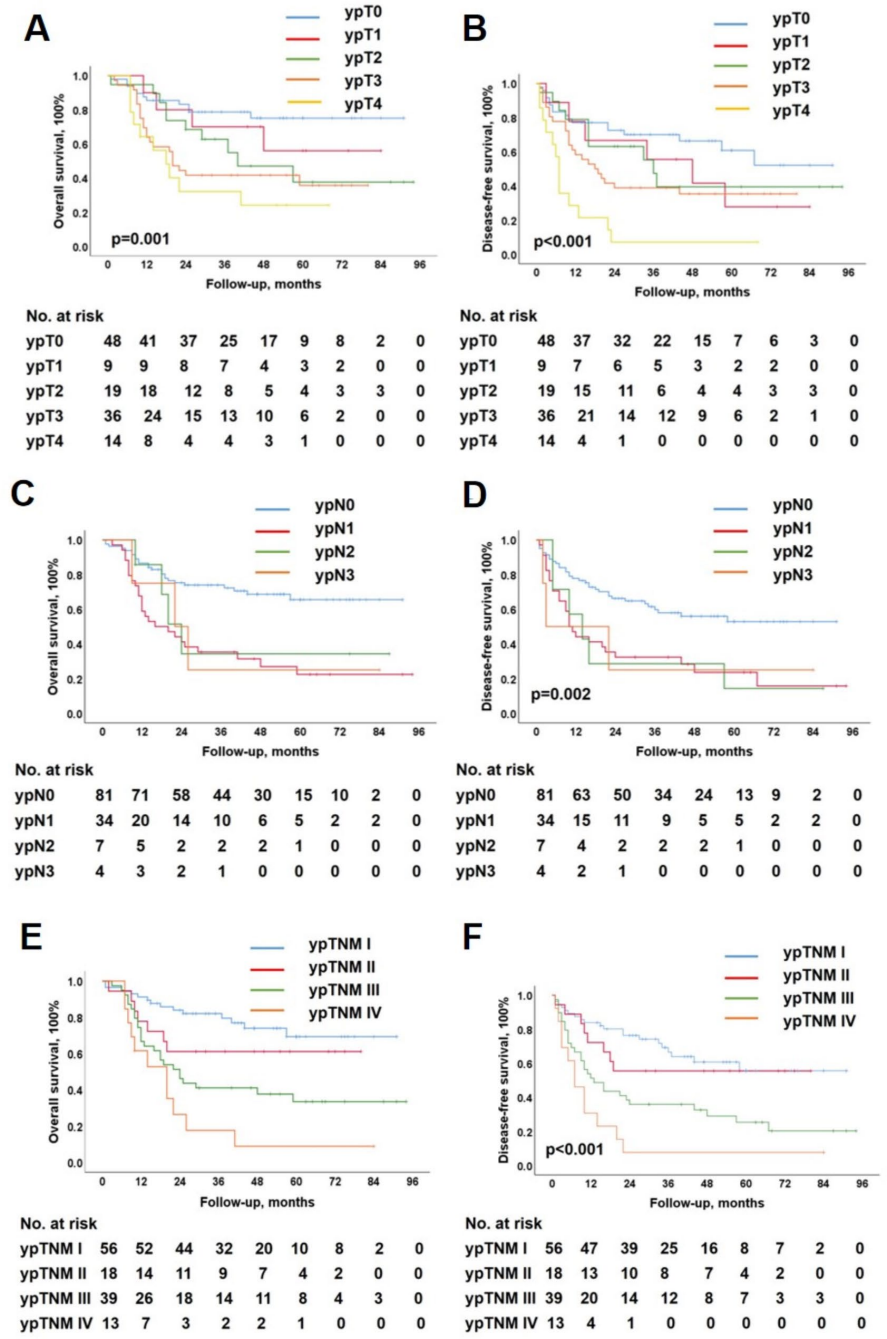
Long-term survival according to different pathological stage: ypT, ypN and ypTNM stage

Survival was calculated to determine the prognostic significance of pCR, MPR, TRG grade, and ypT, ypN, and ypTNM stages. Figures 2 and 3 show the Kaplan–Meier curves for the six pathological statuses.

Compared to the non-pCR patients, the pCR patients had a better 5-year OS (81.2% vs. 39.2%, *p* < 0.001; Fig. 2A). The MPR patients had better 5-year OS than non-MRP patients (64.8% vs. 31.5%, *p* < 0.001; Fig. 2C). The TRG1, TRG2, TRG3, TRG4, and TRG5 groups had 5-year OS of 75.3%, 47.9%, 38.9%, 19.0%, and 35.4%, respectively (*p* = 0.001; Fig. 2E). The ypT0, ypT1, ypT2, ypT3, and ypT4 groups had 5-year OS of 75.1%, 56.0%, 37.6%, 35.7%, and 24.1%, respectively (*p* = 0.001; Fig. 3A). The ypN0, ypN1, ypN2, and ypN3 groups had 5-year OS of 65.5%, 22.4%, 34.3%, and 25.0%, respectively (*p* < 0.001; Fig. 3C). The ypstage I, II, III, and IV groups had 5-year OS of 69.3%, 61.1%, 33.4%, and 8.8%, respectively (*p* < 0.001; Fig. 3E).

Similarly, compared to the non-pCR patients, the pCR patients had better 5-year DFS (69.6% vs. 31.0%, *p* < 0.001; Fig. 2B). The MPR patients had better 5-year DFS than non-MRP patients (51.6% vs. 27.9%, *p* < 0.001; Fig. 2D). The TRG1, TRG2, TRG3, TRG4, and TRG5 groups had 5-year DFS of 53.8%, 34.9%, 37.5%, 19.0%, and 27.6%, respectively (*p* = 0.003; Fig. 2F). The ypT0, ypT1, ypT2, ypT3, and ypT4 groups had 5-year DFS of 60.8%, 27.8%, 39.5%, 35.4%, and 7.1%, respectively (*p* < 0.001; Fig. 3B). The ypN0, ypN1, ypN2, and ypN3 groups had 5-year DFS of 52.9%, 15.7%, 14.3%, and 25.0%, respectively (*p* = 0.002; Fig. 3D). The ypstage I, II, III, and IV groups had 5-year DFS of 55.7%, 55.6%, 25.4%, and 7.7%, respectively (*p* < 0.001; Fig. 3F).

### Cox regression analysis for survival

The prognostic significance of different pathological statuses in the Cox regression analyses is shown in Table 3. Multivariate analysis revealed that the ypT and rTN stages (*p* = 0.026, 0.001) and the ypTNM stage (*p* = 0.010) were independent prognostic factors for OS and DFS, respectively.

**Table 3:** Cox regression analysis for overall survival and disease-free survival

	Univariate analysis		Multivariate analysis	
	*P* value	HR (95%CI)	*P* value	HR (95%CI)
Overall survival				
Pathological complete response, pCR	0.001	0.232 (0.100-0.541)		
Major pathological response, MPR	< 0.001	0.379 (0.222–0.647)		
ypT stage	< 0.001	1.488 (1.231–1.799)	0.026	1.284 (1.030–1.600)
ypN stage	0.001	1.582 (1.210–2.069)		
ypTNM stage	< 0.001	1.819 (1.420–2.331)		
TRG grade	< 0.001	1.371 (1.168–1.608)		
rTN stage	< 0.001	1.731 (1.382–2.169)	0.001	1.527 (1.186–1.966)
Disease free survival				
Pathological complete response, pCR	0.001	0.330 (0.168–0.645)		
Major pathological response, MPR	0.001	0.431 (0.267–0.696)		
ypT stage	< 0.001	1.404 (1.181–1.669)		
ypN stage	0.001	1.552 (1.200-2.008)		
ypTNM stage	< 0.001	1.733 (1.381–2.174)	0.010	1.677 (1.040–2.936)
TRG grade	< 0.001	1.318 (1.140–1.523)		
rTN stage	< 0.001	1.555 (1.272–1.902)		

## Discussion

In this retrospective cohort study, we found that 28.7% of patients after NCRT acquired pCR (ypT0N0), with a 5-year OS of 81.6%, significantly better than non-pCR patients. Among all the patient groups, the poorest survival was observed in patients exhibiting the pathological status of ypT + N+ (27.2%). Meanwhile, the patients who exhibited the ypT + N0/ypT0N + pathology status (44.1%) showed similar survival rates. The independent prognostic value of the revised TN stage according to the pathological status after NCRT was further verified by Cox regression analysis.

Although pCR maybe not the best substitute for OS, its pathological status is closely associated with patients’ prognosis [5]. The 5-year OS was 62.0-79.3% for esophageal cancer patients with pCR after NAT in previous studies [6, 7, 12–14], better than those patients without pCR no matter what kind of NAT strategies used. In the present study, 28.7% of patients acquired pCR, with a 5-year OS of 81.6%. However, the present study showed a high risk of recurrence (26.3%) even after pCR, similar to previous reports of 15.6–25% [14–16].

In clinical practice, we should approached with caution. Currently, pCR is defined as complete tumor regression in surgical specimens, including primary tumors and LNs. If the metastatic LNs were not completely removed, it would inevitably affect the evaluation of the results. Going one step further, there was still tumor residue in the patients’ body and complete remission was not achieved by NAT, even after the complete removal of the primary lesion and LNs. In the NEOCRTEC 5010 study, patients who underwent NCRT exhibited 33.7% recurrence rate (9.8% local and 19.6% distant) after 38.4 months of median follow-up period [4].

A higher pCR was not be translated into survival benefits in previouos studies. In the Japanese JCOG1109 study [17] and the Chinese 1701 study [18], NCRT achieved better pCR but did not yield survival benefits compared with neoadjuvant chemotherapy. Additionally, even for patients with pCR brought about by different neoadjuvant therapies, the survival rates were not consistent [19]. Consequently, exploration of effective NAT strategies is essential to decrease metastasis and recurrence rates and increase the pCR rate following surgery.

The number of ypT + N0 patients (36.0%) in present study was the largest, whereas the number of ypT0N + patients (8.1%) was the smallest, similar to previous analyses [13, 20]. The pathological ypT0N + was unusual after NCRT, ranging from 4–16.3% [6, 13, 20]. Several factors like radiation dose and field, NAT regimen, and aggressive LN dissection may lead to variations in the incidence of ypT0N +. As mentioned, incomplete LN dissection may lead to the classification of ypT0N + patients as having pCR.

Interestingly, the survival between the ypT + N0 and ypT0N + groups was almost the same, worse than that of the ypT0N0 patients, and better than that of the ypT + N + patients. A previous study reported that the ypT0N0, ypT + N0, ypT0N+, and ypT + N + patients had 5-year OS of 62%, 49%, 47%, and 22%, respectively (*p* < 0.001)^13^. By combining the ypT0N + and ypT + N0 with comparable survival outcomes into a “Mid” category, they obtained 5-year OS rates of 69.5%, 40.6% and 31.0% for ypT0N0 (High), Mid and ypT + N+ (Low) group, respectively [7]. Park et al. [6] also reported that the OS of ypT0N + was comparable to ypT + N0 but lower than pCR, with ypT0N + showing similar rates of locoregional and distant metastasis to those of ypT + N0. Contrastingly, we found that ypT0N + patients showed similar locoregional recurrence but more distant metastases than ypT + N0 patients, which may be attributed to the limited case sample or the more aggressive ypT0N + phenotype with LN metastasis.

LN status following NCRT can serve a predictive factor for survival. Compared to ypT + N0, ypT0N + has been reported to have a worse prognosis, which is comparable to ypT + N + ^20^. Schroeder et al. [14] analyzed the data of 201 patients demonstrated pCR of primary tumor and the 5-year OS of ypT0N0 was markedly higher compared to that of ypT0N+ (77% vs. 24%; *p* < 0.0001), suggesting that complete and aggressive LN dissection is crucial, even for patients undergoing NCRT, as it can accurately assess the LN status and improve patient survival. Guo et al. [21]. used 20 LNs to confirm the criteria for LN dissection extent and reported that dissection of > 20 nodes can be an independent predictor of patient survival after NCRT. Chao et al. [22]. reported similar results in ypT0N0 patients after NCRT. In our cohort, 21 LNs were dissected on an average, with the ypT + N + group demonstrating a higher count of harvested LNs (*p* = 0.087).

Our analysis identified various differences in the survival data of our findings and the 8th edition AJCC ypStage. First, although ypT0N0 and other ypTis-2N0 patients are categorized as ypStage I according to the 8th edition AJCC system, they showed varied survival outcomes, with ypT0N0 showed significantly better survival than the other stages. Second, the AJCC system categorizes ypT1-2N0 and ypT0N1-2 as yp Stage I and yp stage III, respectively; however, the two groups showed no significant difference in the survival outcomes. Similar results were reported in another study [6]. These findings highlight concerns about the reliability of the current 8th edition AJCC staging system in predicting patient prognosis following NAT and surgery. The rTN stage was confirmed as an independent predictor of overall survival by Cox regression analysis. The modified TRG based on the pathological status of the primary tumor and LNs indicated better discrimination ability and prediction accuracy that the ypTNM staging system [7]. Overall, ypT0N0 patients showed improved survival compared to ypT + N0 patients, but the current AJCC system identified ypT0N + stage to be higher than the ypT + N0 stage.

### Limitations

Selection bias must be considered in single-center retrospective studies. In addition, this study enrolled fewer patients, especially in the ypT0N + subgroup (11 patients) necessitating a subsequent multicenter collaborative study using a sufficiently large sample size to obtain a large-scale independent dataset for external validation. Due to possible changes in the treatment policies during our study period, several patients may have undergone MIEs. We maintained uniform radiotherapy and surgical policies throughout the study period, primarily focusing on ESCC, excluding esophageal adenocarcinoma. However, our study identified various significant findings on the survival of ESCC patients after NCRT and surgery. Additionally, it highlighted the discrepancies between the 8th edition AJCC ypStage grouping and ground-truth data.

## Conclusions

The present study found that pCR (ypT0N0) after NCRT was associated with prolonged survival in patients with ESCC; however, the presence of residual primary lesions and nodal metastases significantly decreased the survival rate of the ESCC patients. We also found the discrepancies identified between the AJCC 8th ypStage grouping and real-world data. In AJCC 8th edition ypStage I patients, ypT0N0 exhibited improved survival outcomes compared to other ypTis-2N0 patients, and pCR was grouped separately from the other ypStage I patients. The ypT0N + and ypT + N0 patients showed similar survival outcomes.

## Data Availability

The datasets used and/or analysed during the current study are available from the corresponding author on reasonable request.
